# LETTER TO THE EDITOR An Update: Does the Type of Skin Marker Prevent Marking Erasure of Surgical-Site Markings?

**Published:** 2010-06-30

**Authors:** Daniel Marsland, Megan Y. Kamath, Simon C. Mears, Stephen M. Belkoff

**Affiliations:** International Center for Orthopaedic Advancement, the Department of Orthopaedic Surgery, The Johns Hopkins University/Johns Hopkins Bayview Medical Center, Baltimore, Maryland

Dear Sir,

We wish to provide readers with an update on an article published in *Eplasty* in 2009.^1^ Our original article reported the performance of commercially available skin marker pens after application of a chlorhexidine-based skin preparation and compared those performances with that of traditional marker pens. Preoperative skin preparation with chlorhexidine-based solution is preferred over iodine-based solution for preventing surgical-site infection.^2^ However, traditional skin marker pens are erased significantly more after application of chlorhexidine-based solution than with iodine-based solution.^1^ Since our previous publication, new pens claiming resistance to erasure have been marketed.

In the current communication, we report on the ability of the new pens to withstand exposure to chlorhexidine-based preparation compared with that of a traditional marker. We also report if marks made with the new pens performed similarly when exposed to chlorhexidine-based solution versus iodine-based solution.

We repeated the protocol of our previous studies^1^,^3^ using 19 fasciocutaneous skin flaps harvested from fresh white cadavers obtained from the State Anatomy Board. One skin marker (Sandel 4-in-1 Marker [skin, wide]; Sandel Medical Industries, LLC, Chatsworth, Calif) was arbitrarily chosen as a traditional marker pen to be compared with 2 new pens advertised as providing skin markings resistant to chlorhexidine-based skin preparation (Viscot 1450XL-200 Mini XL presurgical skin marker, fine tip, and Viscot 1449XL-50 XL presurgical skin marker, bold tip; Viscot Medical, LLC, East Hanover, NJ). Using Adobe Photoshop CS2 (Adobe Systems, Inc, San Jose, Calif), we measured the marker-to-skin contrast before and after application of the solution. Differences were checked for significance (*P* < .05).

After application of the chlorhexidine-based solution, marks made with the new pens resisted erasure significantly better than did marks made with the traditional pens (Table 1, Fig 1). We observed no significant differences in erasure of markings between the 2 new markers after application of the chlorhexidine- or iodine-based solutions. Significant erasure occurred for all skin markings after application of the chlorhexidine-based solution relative to the preapplication markings and compared with mark erasure observed after application of the iodine-based solution (Table 1).

The marks made by using the Viscot Mini XL and XL bold pens remained significantly more visible than those made with the traditional pen after application of the chlorhexidine-based solution. However, markings from both new pens were still erased significantly by application of the chlorhexidine-based solution. To our knowledge, there are no standards regarding the amount of erasure permitted; however, any erasure could compromise patient safety. It is currently unknown how much marker erasure is attributed to the scrubbing process required for the chlorhexidine-based solution. The ink used in the Viscot markers in our study is proprietary, so we do not know what particular component of the ink improved its resistance to the chlorhexidine-based solution.

Although the new marker withstood marking erasure better than did previously investigated pens, improvements are still needed. Such improvements could come from development of additional pens and changes in prepared solution or its application.

## Figures and Tables

**Figure 1:**
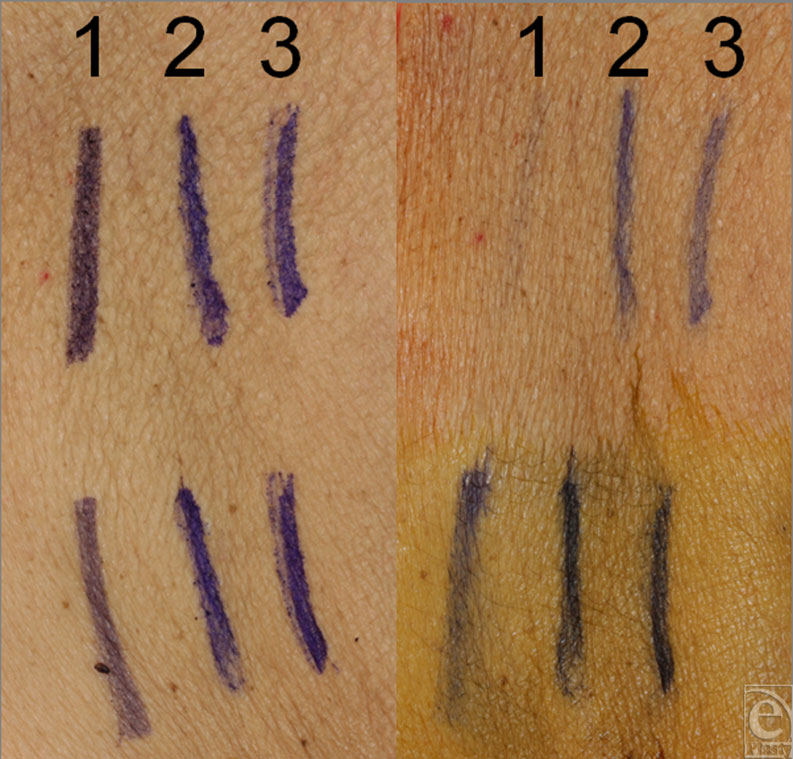
Photographs of skin markings before (*left*) and after (*right*) the application of a chlorhexidine-based (*top*) or iodine-based (*bottom*) skin preparation solution. Marks made with each of the pens from left to right are numbered as follows: (1) Sandel 4-in-1, (2) Viscot Mini XL, and (3) Viscot XL bold.

**Table 1:** Mean change in marker-to-skin contrast by skin preparation*

	Mean difference (95% CI)	
Type of pen	Chlorhexidine solution	Iodine solution
Sandel 4-in-1	42.2 (36.1-48.4)	1.8 (−3.5 to 7.0)
Viscot Mini XL	27.4 (22.4-32.4)	14.23 (10.9-17.5)
Viscot XL bold	26.0 (22.0-30.1)	17.0 (13.6-20.4)

*Relatively larger values indicate loss of marker-to-skin contrast, that is, more marking was erased.
